# Metabolic syndrome among people living with HIV in Ethiopia: a systematic review and meta-analysis

**DOI:** 10.1186/s13098-023-01034-9

**Published:** 2023-03-28

**Authors:** Derara Girma, Hiwot Dejene, Leta Adugna Geleta, Erean Shigign Malka, Mengistu Tesema, Mukemil Awol, Befekadu Tesfaye Oyato

**Affiliations:** 1 Public Health Department, College of Health Sciences, Salale University, Fiche, Ethiopia; 2 Department of Midwifery, College of health sciences, Salale University, Fiche, Ethiopia

**Keywords:** Metabolic syndrome, HIV/AIDS, Ethiopia, Meta analysis

## Abstract

**Background:**

Human Immuno-deficiency Virus (HIV) infection and antiretroviral therapy (ART) can cause metabolic disorders such as lipodystrophy, dyslipidemia, and insulin resistance, all of which are symptoms of metabolic syndrome (MetS). In Ethiopia, despite the existence of the primary studies, there was no pooled study conducted to summarize the country-level MetS among people living with HIV (PLHIV). Therefore, this study aims to estimate the pooled prevalence of MetS among PLHIV in Ethiopia.

**Methods:**

A systematic search was conducted to retrieve studies on the prevalence of MetS among PLHIV in Ethiopia from PubMed, Google Scholar, Science Direct, Web of Sciences, HINARI, and other relevant sources. A random-effects model was used to estimate the MetS in this study. The overall variation between studies was checked by the heterogeneity test (I^2^). The Joanna Briggs Institute (JBI) quality appraisal criteria were used to assess the quality of the studies. The summary estimates were presented with forest plots and tables. Publication bias was checked with the funnel plot and Egger’s regression test.

**Results:**

Overall, 366 articles were identified and evaluated using the PRISMA guidelines, with 10 studies meeting the inclusion criteria included in the final analysis. The pooled prevalence of MetS among PLHIV in Ethiopia was 21.7% (95% CI:19.36–24.04) using National Cholesterol Education Program Adult Treatment Panel III (NCEP/ATP III) and 29.91% (95% CI: 21.54–38.28) using International Diabetes Federation (IDF) criteria. The lowest and highest prevalence of MetS were 19.14% (95%CI: 15.63–22.64) and 25.6% (95%CI: 20.18–31.08) at Southern Nation and Nationality People Region (SNNPR) and Addis Ababa, respectively. There was no statistical evidence of publication bias in both NCEP-ATP III and IDF pooled estimates.

**Conclusion:**

MetS was common among PLHIV in Ethiopia. Therefore, optimizing regular screening for MetS components and promoting a healthy lifestyle is suggested for PLHIV. Furthermore, more study is contributory to identify the barriers to implementing planned interventions and meeting recommended treatment goals. **Trial registration: **The review protocol was registered in the International Prospective Register of Systematic Reviews (PROSPERO): CRD42023403786.

**Supplementary Information:**

The online version contains supplementary material available at 10.1186/s13098-023-01034-9.

## Background

World Health Organization (WHO) estimated that HIV/AIDS remains a major global public health concern, having claimed the lives of 40.1 million [33.6–48.6 million] people to date. In 2021, HIV-related causes killed 650,000 [510,000-860,000] people, and 1.5 million [1.1-2.0 million] were newly infected. Furthermore, approximately 38.4 million [33.9–43.8 million] people were living with HIV at the end of 2021, with the WHO African Region accounting for two-thirds (25.6 million) of the total [1]. During the same year, about 75% of all PLHIV were accessing treatment. In the East and Southern African regions, 78% of PLHIV receive treatment. Subsequently, the availability of ART and effective tools for preventing, detecting, and treating opportunistic infections (OIs) is significantly reducing HIV-related deaths [2].

With the expansion of effective ART, life expectancy and quality of life of PLHIV have improved considerably. This is due to the marked advances in its potency, low adverse effect, and simplicity of the usage [3]. However, longer life expectancy of PLHIV and long-term use of combined ART have raised the concerns about increased cardiovascular risk, which are frequently grouped together and manifested as MetS [4]. Specifically, protease inhibitors (PIs) containing regimens are associated with increase in serum lipid levels and increased risk of dyslipidemia [4, 5]. On the other hand, through chronic inflammation and immune dysfunction, the virus itself can increase the risk of dyslipidemia, atherosclerosis, and type two diabetes mellitus (T2DM) [6].

MetS is a cluster of risk factors that includes factors like raised blood sugar, high blood pressure, dyslipidemia, and abdominal obesity. It is a common disorder that is associated to an increased risk of cardiovascular disease (CVD), and T2DM in both genders [7]. Aside from the cardiovascular consequences, MetS patients are considered to be more vulnerable to a variety of conditions. These include, vascular diseases (e.g., atherosclerotic cardiovascular disease and hypertension), adiposity-related disorders (e.g., sleep disordered breathing and fatty liver disease), insulin resistance conditions (e.g., T2DM or gestational diabetes and polycystic ovary syndrome), atherogenic dyslipidemia, hormonal dysfunction, and chronic kidney disease [8]. Particularly, the consequences of MetS are much aggravated among PLHIV and receiving ART [6, 9–11].

A comprehensive review and meta-analysis of MetS prevalence in the global HIV-infected population revealed a high burden of MetS ranging from 16.7 to 31.3% depending on the definition used. It was also noted that the wide prevalence range reflects significant heterogeneities across and within the diagnostic criteria [12]. Additionally, MetS was reported in 21.5% of PLHIV in Sub-Saharan African countries [13].

In Ethiopia, different primary studies have reported a prevalence of MetS among PLHIV ranging from 16.8 to 43.8% based on various criteria [14–23]. Despite the fact that a number of primary studies have been conducted to assess the prevalence of MetS, no systematic review and meta-analysis (SRMA) has been conducted to synthesize this evidence in order to better understand the disease’s occurrence at the national level. Also, pooling the prevalence of MetS among PLHIV will enable to overcome the existing discrepancies and will support in developing of preventive and management strategies for healthcare services. Therefore, this SRMA was conducted to estimate the pooled prevalence of MetS among PLHIV in Ethiopia.

## Methods

### Protocol registration

The review protocol was registered in the International Prospective Register of Systematic Reviews (PROSPERO). This study’s protocol can be accessed via a web address (https://www.crd.york.ac.uk/PROSPERO/#myprospero). The protocol registration number is CRD42023403786.

### Search strategy

To conduct this SRMA different electronic databases such as PubMed, Science Direct, Web of Sciences, World cat, DOAJ, and Hinari were systematically searched. Also, other sources such as Google Scholar, GOOGLE, journal homepages, institutional repositories, and bibliographies were searched to retrieve eligible studies. The search terms were used as keywords and MeSH terms both individually and in conjunction with the “OR” and “AND” Boolean operators. Consequently, “Acquired Immunodeficiency Syndrome“[Mesh] OR HIV/AIDS[tiab] OR “HIV“[Mesh] OR “HIV positive“[tiab] OR “people live with HIV“[tiab] AND (“Metabolic Syndrome“[Mesh] OR “Metabolic syndrome“[tiab] OR “cardiometabolic syndrome“[tiab] OR “Metabolic Diseases“[Mesh] AND Ethiopia were used. The EndNote X7 software was used to manage the search results. Moreover, the Preferred Reporting Items for Systematic Review and Meta-Analysis (PRISMA) guideline was used to design and report the findings of this study [24].

### Eligibility criteria

#### Inclusion criteria

This SRMA included observational studies conducted in Ethiopia among adult PLHIV aged ≥ 18 years and accessible before September 17, 2022. Furthermore, only original full-text articles and studies written in English language were included.

#### Exclusion criteria

Studies that did not report prevalence of MetS, measured different outcomes, and did not include IDF or NCEP/ATP III criteria were excluded.

### Outcome measurement

The outcome variable in this study was MetS in PLHIV, which was defined using NCEP-ATP III or IDF criteria.

#### NCEP-ATP III definition

Any three of the following five criteria (no absolutely required criterion) [8, 25].

#### IDF definition

Central obesity (waist circumferences of 94 cm for men and 80 cm for women) combined with two of the four criteria listed below [8, 25].

**Criteria**

- Obesity: Waist circumference of > 102 cm for men and > 88 cm for women.
- Hyperglycemia: Fasting glucose > 100 mg/dl or pharmacologic treatment.
- Dyslipidemia: Triglyceride (TG) > 150 mg/dl or pharmacologic treatment.
- Dyslipidemia (second, separate criteria): high-density lipoprotein cholesterol (HDL-C): <40 mg/dl for males and < 50 mg/dl for females or pharmacologic treatment.
- Hypertension: systolic blood pressure > 130 mmHg or diastolic blood pressure > 85 mmHg or pharmacologic treatment.

### Data extraction

The data from each study were extracted by two independent authors (DG and HD) with a customized format in Microsoft Excel sheet. The disagreements between the two independent authors were resolved by the other authors. The extracted data included; Author names, year of publication, sample size, study design, region, and settings in which the studies were conducted.

### Quality and risk of bias assessment in individual studies

The Joanna Briggs Institute (JBI) tool for cross-sectional studies [26] was used to assess the methodological quality and risk of bias (in design, conduct, and analysis) of included studies (supporting file 1). The tool was used to evaluate the inclusion criteria, measurement of outcome variables, confounding adjustment, and statistical analysis appropriateness. The quality of the extracted studies was evaluated by all authors who participated in data extraction.

### Data analysis and synthesis

The extracted data were exported to STATA-14 for analysis. The pooled prevalence of MetS was determined with a random effect model as there was substantial heterogeneity. The heterogeneity among the included studies was checked by forest plot, Cochran’s Q (χ^2^ test), I^2^ test, and p-value. The heterogeneity was considered as low, moderate or high when I^2^ test statistics results were 25%, 50%, and 75% respectively [27]. Forest plots were also used to visualize the presence of heterogeneity. Sub-group analysis and meta-regression were also performed to identify the source of heterogeneity. Publication bias was checked using funnel plot of symmetry. Further, the statistical significance of publication bias was checked using Egger and Begg tests [28]. A p-value less than 0.05 was used to declare the presence of publication bias. Sensitivity analysis using a random effects model was computed to assess the influence of a single study on the overall meta-analysis estimate.

## Results

### Search results

**Fig. 1:**
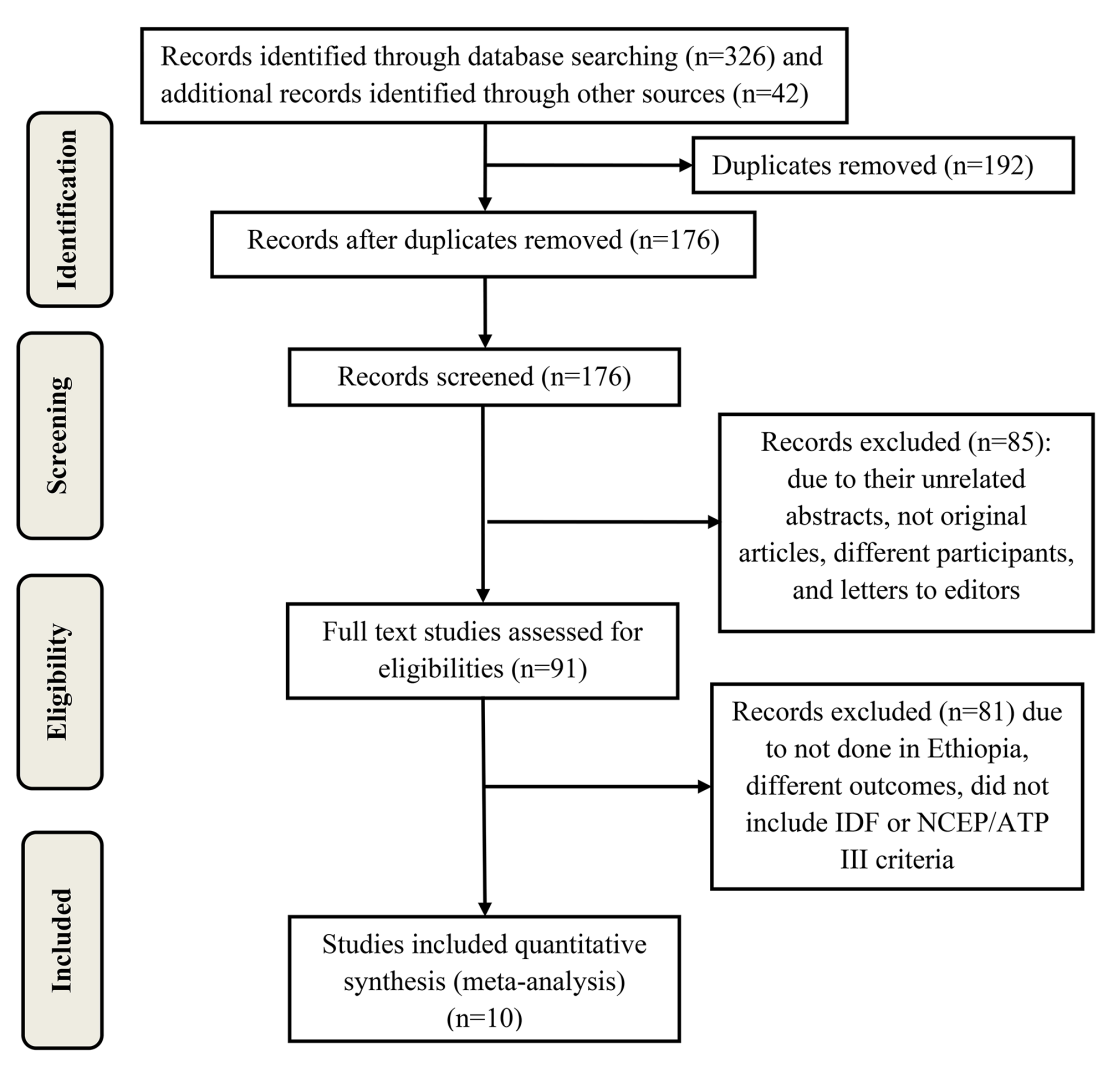
PRISMA 2020 flow diagram showing the selection process of studies in the systematic review and meta-analysis

Initially, 366 studies were retrieved by different search techniques. One hundred and ninety-two studies were excluded because of duplication. Among the remaining 176 studies, 85 were excluded due to their unrelated abstracts, not original articles, different participants, and letters to editors. Other 81 studies were also excluded because they were not done in Ethiopia, had different outcomes, and did not include IDF or NCEP/ATP III criteria. Finally, 10 studies that met the inclusion criteria were included in the final SRMA (Fig. 1).

### Study characteristics

This SRMA comprised 10 studies done in Ethiopia with a total of 3,751 subjects. Of the total study subjects, female patients were 2,380 (63.5%). Regarding the study regions, four studies were conducted in SNNPR [14–17], two studies were in Oromia region [18, 19], two studies were in Addis Ababa [20, 21], one study was in Amhara region [22], and one study was in Harari region [23]. Cross-sectional study designs were used in all included studies and all of them were published articles. Furthermore, all studies were published between 2012 and 2022, with sample sizes ranging from 185 to 633, and included both sexes as study participants. The prevalence of MetS was assessed using the NCEP/ATPIII and IDF criteria. Accordingly, eight studies [16–21, 23] used NCEP/ATPIII, six studies [14, 16–18, 20, 22] used IDF, and four studies used both tools [15–18, 20] (Table 1).

**Table 1:** Characteristics of the included studies

Author (year)	Region	Study setting	Study design	Sample size	Sampling technique	Sex of the participants	MetS. % (NCEP-ATP III)	MetS % (IDF)
Woldu et al. (2022) (20)	Addis Ababa	Zewditu memorial hospital	Cross-sectional	288	Probability	Both men and women	28.5	43.8
Woldeyes et al. (2022) (21)	Addis Ababa	SPHMMC	Cross-sectional	275	Probability	Both men and women	22.9	-
Tesfaye et al. (2014) (17)	SNNPR	Hawassa Univ. Ref. Hospital	Cross-sectional	374	Probability	Both men and women	16.8	23.8
Hirigo et al. (2016) (16)	SNNPR	Hawassa Univ. Ref. Hospital	Cross-sectional	185	Probability	Both men and women	17.8	24.3
Gebrie (2020) (22)	Amhara	Debre M. /fel. hiw Hospital	Cross-sectional	407	Probability	Both men and women	-	24.6
Bune et al. (2020) (14)	SNNPR	Gedeo zone PH facilities	Cross-sectional	633	Non-probability	Both men and women	-	42.5
Bosho et al. (2018) (18)	Oromia	Jimma PH facilities	Cross-sectional	268	Probability	Both men and women	23.5	20.5
Berhane et al. (2012) (19)	Oromia	Jimma univ. spec. hosp.	Cross-sectional	313	Non-probability	Both men and women	21.1	-
Ataro et al. (2020) (23)	Harari	Jugal Hospital	Cross-sectional	375	Non-probability	Both men and women	22.1	-
Bune et al. (2020) (15)	SNNPR	Gedeo zone PH facilities	Cross-sectional	633	Non-probability	Both men and women	22.0	-

NCEP-ATP III = National Cholesterol Education Program Adult Treatment Panel III; IDF = International Diabetes Federation; JBI = Joanna Briggs Institute; SNNPR = Southern Nation and Nationalities People Region; SPHMMC = Saint Paul Hospital Millennium Medical College

### Components of metabolic syndrome

The prevalence of the individual components of MetS varied significantly between studies in the Ethiopian PLHIV. The overall existences of the components were; 47.28% (95%CI: 33.18, 61.38) for low HDL-C, 39.02% (95%CI: 36.07, 41.98) for Elevated triglyceride, 36.36% (95% CI: 23.76, 48.96) for central obesity, 31.78% (95%CI: 17.61, 45.95) for high blood pressure, and 31.39% (95% CI: 22.79, 39.99) for hyperglycemia (Table 2).

**Table 2:** Pooled estimates of MetS components

Author (year)	Components				
	High blood pressure	Hyperglycemia	Low HDL-C	Central obesity	Elevated triglyceride
Woldeyes et al. (2022) (21)	28.36	42.18	12.36	30.91	43.64
Woldu et al. (2022) (20)	-	-	-	-	-
Tesfaye et al. (2014) (17)	19.25	27.54	66.31	50.80	41.18
Hirigo et al. (2016) (16)	9.73	31.35	70.27	51.35	44.86
Gebrie (2020) (22)	-	-	-	41.28	-
Bune et al. (2020) (14)	55.61	56.24	34.28	40.76	37.12
Bosho et al. (2018) (18)	38.43	17.16	49.25	18.66	29.85
Berhane et al. (2012) (19)	35.14	24.92	47.60	6.07	38.98
Ataro et al. (2020) (23)	10.93	25.07	64.53	59.2	41.87
Bune et al. (2020) (15)	56.87	26.38	34.28	28.91	36.97
**Total pooled estimates (95% CI)**	**31.78 (17.61, 45.95)**	**31.39 (22.79, 39.99)**	**47.28 (33.18, 61.38)**	**36.36 (23.76, 48.96)**	**39.02 (36.07, 41.98)**

### Pooled prevalence of metabolic syndrome using NCEP/ATP III

**Fig. 2:**
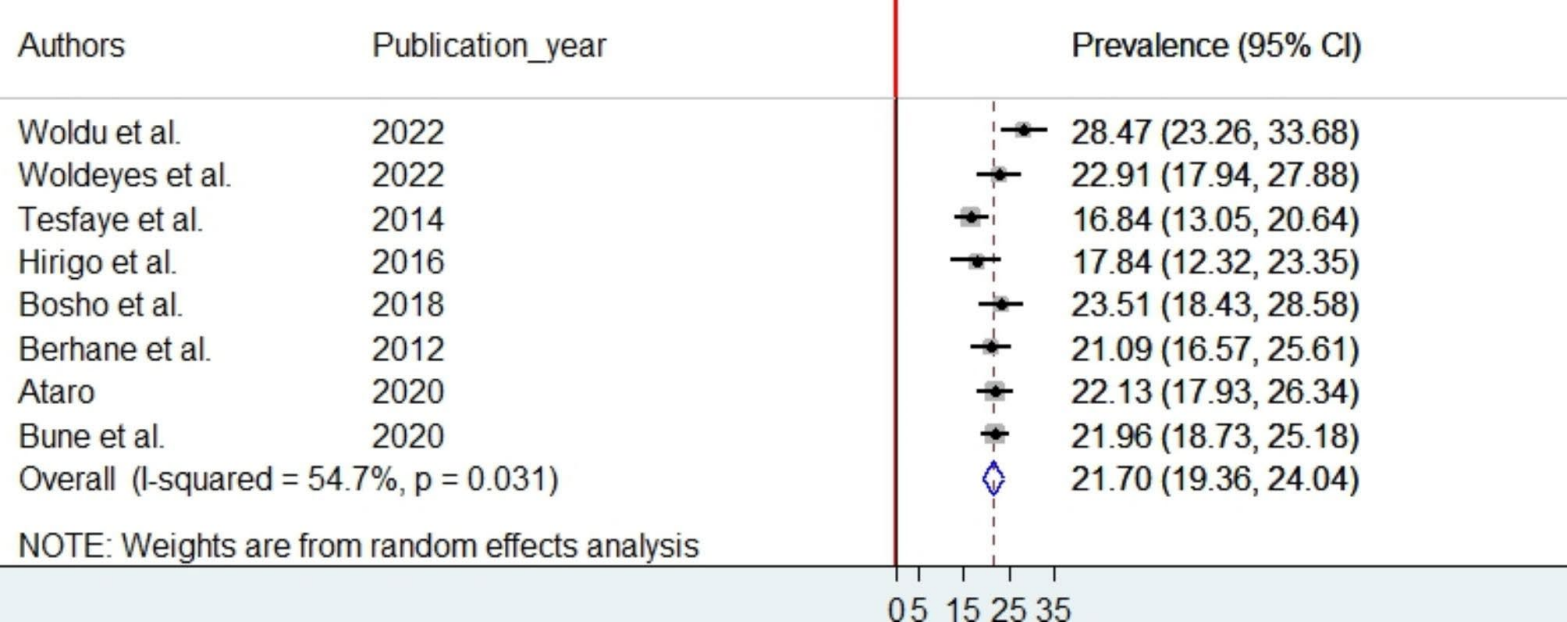
The forest plot of pooled prevalence of MetS according to NCEP-ATP III criteria

**Fig. 3:**
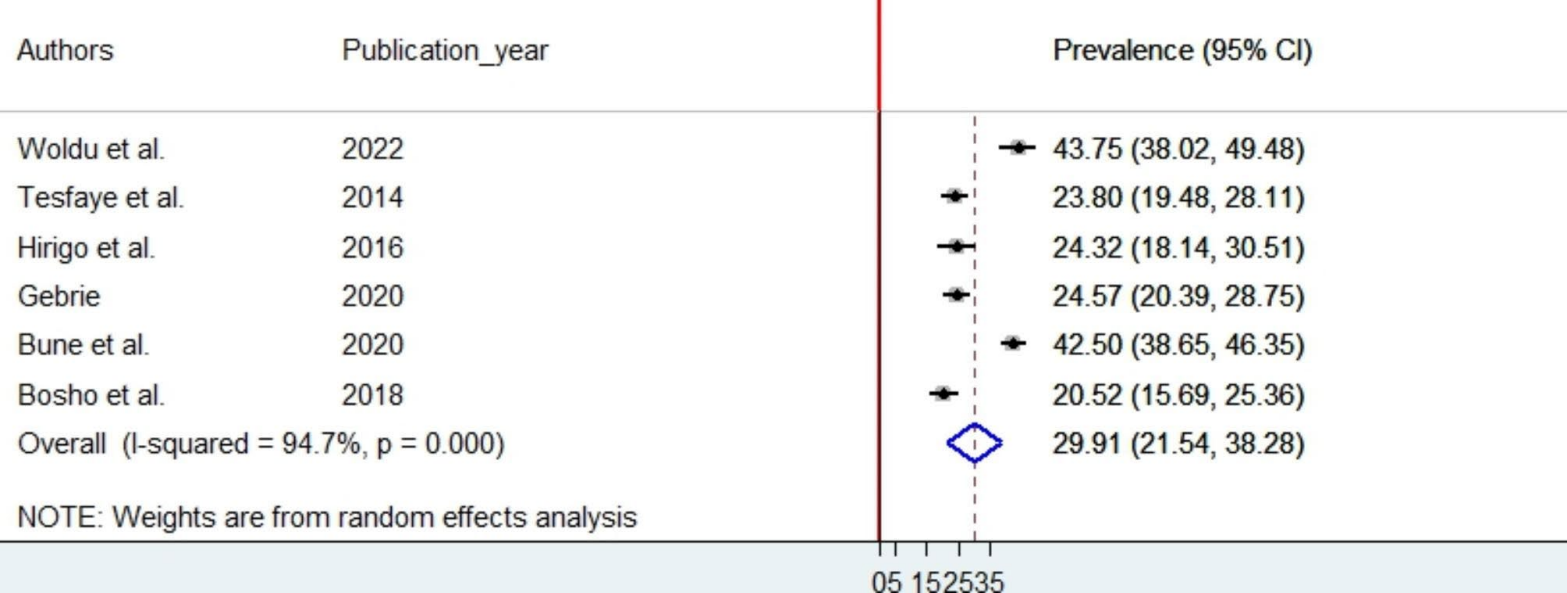
The forest plot of pooled prevalence of MetS according to IDF criteria

The random-effects model was used because considerable heterogeneity was observed across the studies. The pooled prevalence of MetS among PLHIV in Ethiopia is 21.7% (95% CI:19.36–24.04) using NCEP/ATP III (Fig. 2) and 29.91% (95%CI: 21.54–38.28) using IDF criteria (Fig. 3). To identify the potential source of heterogeneity across studies, subgroup analysis based on region and sampling technique was considered. However, the subgroup analysis result indicated that the source of heterogeneity was not due to region and sampling technique differences. The lowest and highest prevalence of MetS were 19.14% (95%CI: 15.63–22.64) and 25.6% (95%CI: 20.18–31.08) at SNNPR and Addis Ababa respectively based on NCEP/ATP III criteria (Supporting file 2). In addition to the subgroup analysis, meta-regression was performed, considering the year of publication and the sample size of the studies. Accordingly, the meta-regression results revealed that heterogeneity in the prevalence of MetS was unrelated to sample size variation (coefficient = -0.0032356, p-value = 0.715) or publication year (coefficient = 0.6811984, p-value = 0.079) based on NCEP/ATP III criteria.

### Publication bias

**Fig. 4:**
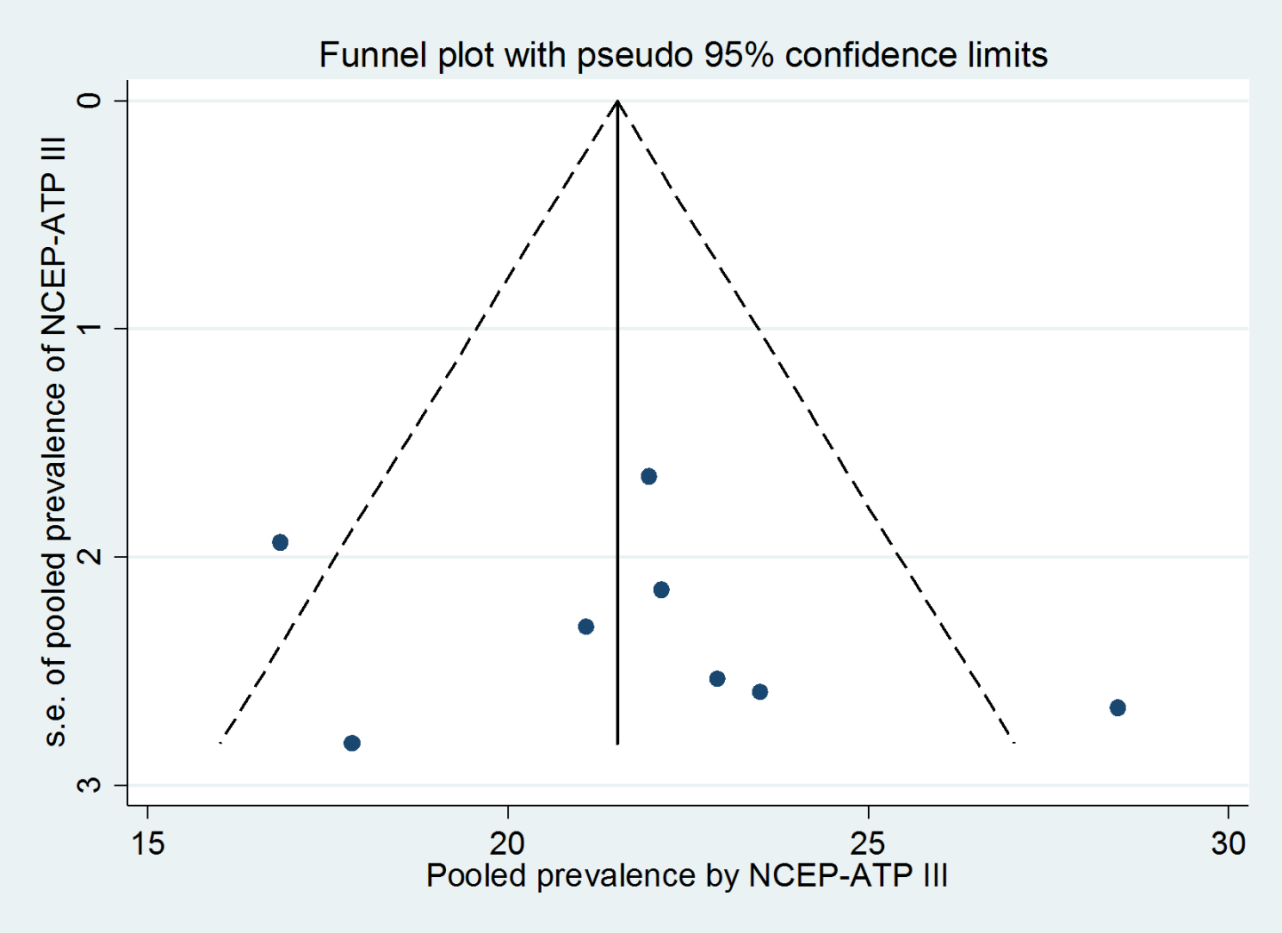
Funnel plots for publication bias based on NCEP-ATP III criteria

**Fig. 5:**
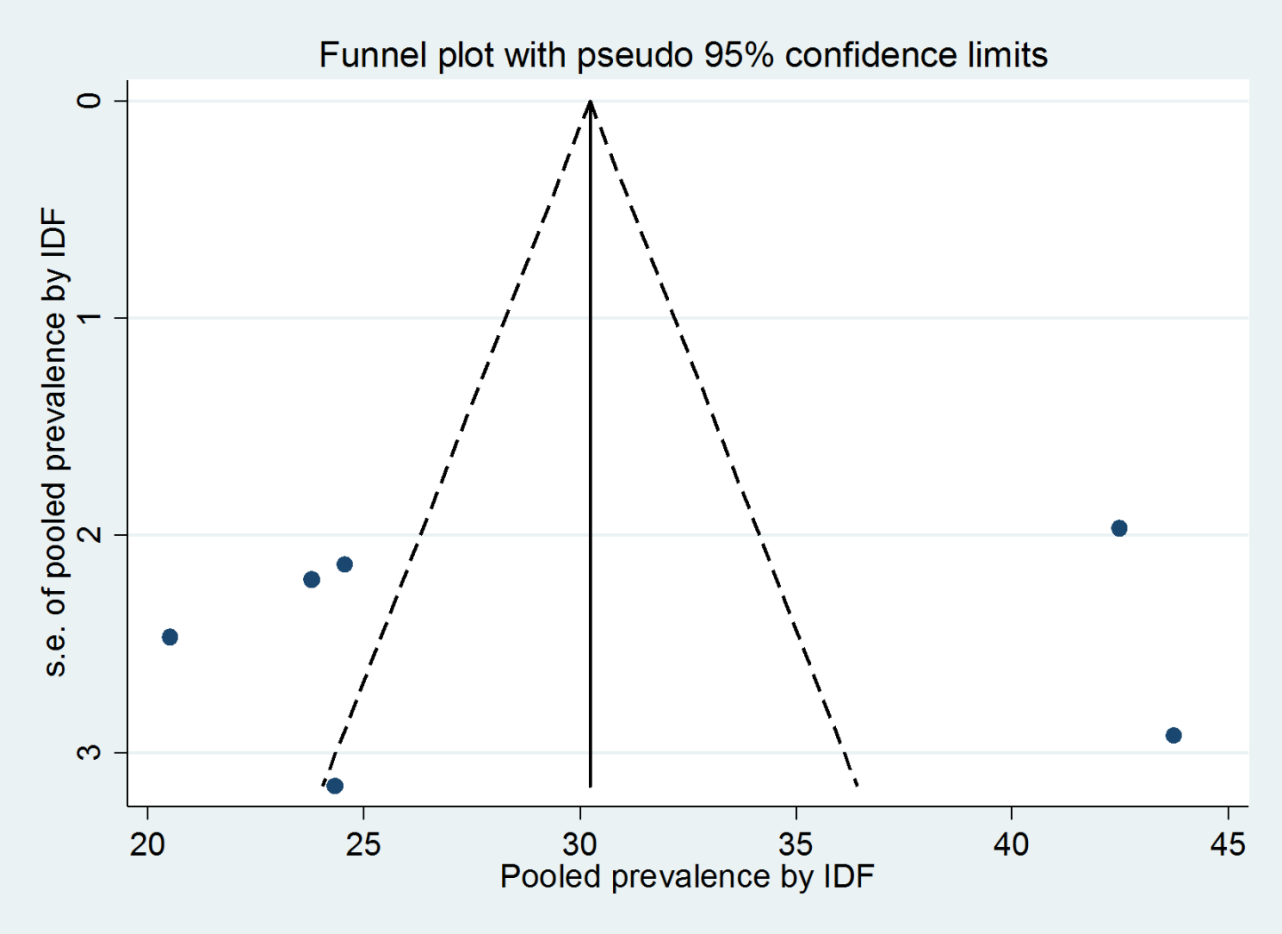
Funnel plots for publication bias based on IDF criteria

The presence of publication bias was assessed using funnel plots and Egger, and Begg statistical tests at 5% significant level. There was no statistical evidence of publication bias in both NCEP-ATP III and IDF pooled estimates. For the NCEP-ATP III pooled estimate, the funnel plot was almost symmetry (Fig. 4), the Begg and Egger tests were statistically non-significant (p-value = 0.266 and p-value = 0.482) respectively. Also, For the IDF pooled estimate, the funnel plot was almost symmetry (Fig. 5), the Begg and Egger tests were statistically non-significant (p-value = 0.707 and p-value = 0.802) respectively.

### Sensitivity analysis

**Fig. 6:**
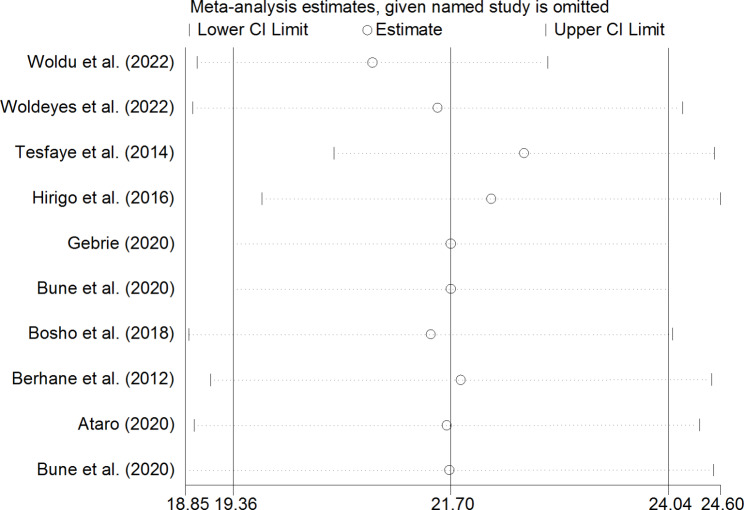
Sensitivity analysis for single study influence of pooled estimate based on NCEP-ATP III criteria

**Fig. 7:**
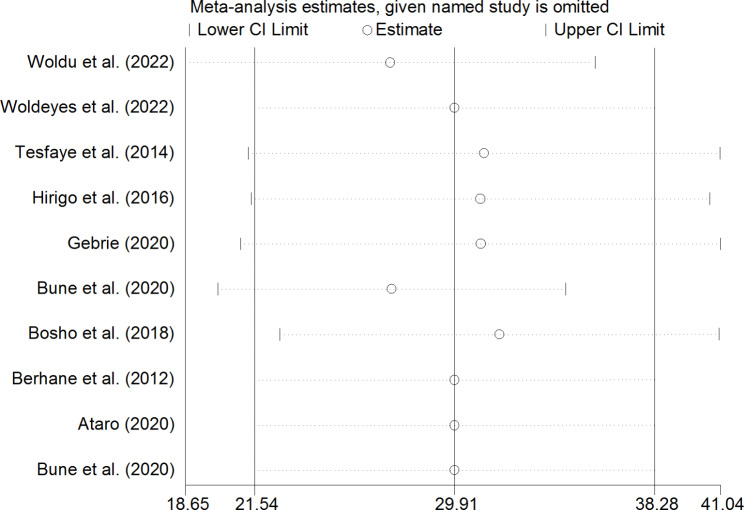
Sensitivity analysis for single study influence of pooled estimate based on IDF criteria

To identify the effect of single study on overall meta-analysis estimate, sensitivity analysis using a random effects model was computed. The analysis found no strong evidence for influence of single study in both NCEP-ATP III (Fig. 6) and IDF (Fig. 7) pooled estimates.

## Discussion

MetS is now becoming a problem for PLHIV [9]. This SRMA disclosed the pooled prevalence of MetS in Ethiopia. Thus, the pooled prevalence of MetS among PLHIV in Ethiopia is 21.7% (95% CI:19.36–24.04) using NCEP/ATP III and 29.91% (95% CI: 21.54–38.28) using IDF criteria. As a result, even though the pooled point prevalence of the MetS is higher with NCEP/ATP III criteria than with IDF criteria, their confidence intervals overlap, indicating the comparability of the criteria. The NCEP/ATP III criteria-based pooled prevalence in this study was consistent with the reports from global SRMA (24.6%) [12], the recent systematic review (20.6%) [29], and the sub-Saharan Africa (SSA) countries (19.9%) [13]. Similarly, the IDF criteria-based pooled prevalence in this study was also comparable with the reports from the sub-Saharan Africa countries (25.7%) [13]. This might be due to use of comparable case definition for MetS. Furthermore, despite an inconsistent depiction of studies from around the world, the prevalence of MetS was roughly similar within and across major regions, including continents and intra-country locations, regardless of the definition criteria used [12].

This finding is also consistent with other SRMA studies conducted in Ethiopia on a diverse range of populations. The SRMA in 2021 found that 30% of people had MetS, with the highest burden among diabetics (56%) and hypertensive patients (44%) [30]. Furthermore, the 2020 SRMA found that the pooled prevalence of MetS in Ethiopia was 34.89% using NCEP/ATP III criteria and 27.92% using IDF criteria [31]. This could be because PLHIV were also a significant part of previous studies, similar diagnosing criteria were used, and the settings were also comparable. Furthermore, the SSA study indicated the need for immediate prevention and management programs to halt the progression of MetS, regardless of HIV status [13].

Low HDL-C was the most common individual component of MetS in the current SRMA which was similar to reports from the SSA countries [13] and Kenya [32]. This suggests that low-HDL continues to be a major precursor for MetS, implying that timely intervention is required to prevent MetS progression. The other MetS component found prevalent in this study was high triglyceride levels. This finding is in agreement with the study from Italy [33]. This is caused not only by the virus, but also by the ART drugs used to treat the infection. According to the most recent large survey, more PLHIV with MetS than those without MetS had ever been exposed to PIs [9]. As a result, a detailed patient’s medical assessment is essential for prioritizing risk mitigation and primary care prevention services.

Furthermore, this SRMA confirms that other major MetS components, such as central obesity, high blood pressure, and hyperglycemia, are predominant among Ethiopian PLHIV. This is evidenced by the previously conducted review article [34]. To prevent CVDs, current HIV recommendations encourage the use of standard lifestyle interventions such as weight loss, exercise, smoking cessation, and eventually pharmacological interventions such as antihypertensive and lipid-lowering therapy, as well as treatment of glucose metabolism alterations [35].

The strengths of this SRMA include a complete literature search in the various relevant database, proper screening of eligible studies, and strong assessment of quality to rule out the quality bias. The limitation was; the meta-analysis result should be interpreted cautiously due to heterogeneity among studies.

## Conclusion

MetS was common among PLHIV in Ethiopia. Therefore, optimizing regular screening for MetS components and promoting a healthy lifestyle is suggested for PLHIV. Furthermore, more study is contributory to identify the barriers to implementing planned interventions and meeting recommended treatment goals. Moreover, a context-specific tool for measuring MetS among PLHIV should be developed to improve the replicability of the studies in Ethiopia.

## Electronic supplementary material

Below is the link to the electronic supplementary material.

Supplementary Material 1

Supplementary Material 2

Supplementary Material 3

Supplementary Material 4

Supplementary Material 5

## Data Availability

The data used to support the findings of this study are included in the article.
