# Supplementary material for: Metabolic syndrome among people living with HIV in Ethiopia: a systematic review and meta-analysis
Source: Diabetol Metab Syndr. 2023 Mar 28;15:61. doi: 10.1186/s13098-023-01034-9 (PMC10045608; doi:10.1186/s13098-023-01034-9)
Supplement: Supplementary file 1 — Supplementary Material 1 [file 13098_2023_1034_MOESM1_ESM.docx]

| Author (year) | Were the criteria for inclusion in the sample clearly defined? | Were the study subjects and the setting described in detail? | Was the exposure measured in a valid and reliable way? | Were objective, standard criteria used for measurement of the condition? | Were confounding factors identified? | Were strategies to deal with confounding factors stated? | Were the outcomes measured in a valid and reliable way? | Was appropriate statistical analysis used? |
| --- | --- | --- | --- | --- | --- | --- | --- | --- |
| Woldu et al. (2022) | Yes | Yes | Yes | Unclear | Yes | Yes | Yes | Yes |
| Woldeyes et al. (2022) | Yes | Yes | Yes | Yes | Yes | Yes | Yes | Yes |
| Tesfaye et al. (2014) | Yes | Yes | Yes | Yes | Yes | Yes | Yes | Yes |
| Hirigo et al. (2016) | Yes | Yes | Yes | Yes | Yes | Yes | Yes | Yes |
| Gebrie (2020) | Yes | Yes | Yes | Unclear | Yes | Yes | Yes | Yes |
| Bune et al. (2020) | Yes | Yes | Yes | Yes | Yes | Yes | Yes | Yes |
| Bosho et al. (2018) | Yes | Yes | Yes | Yes | Yes | Yes | Yes | Yes |
| Berhane et al. (2012) | Yes | Yes | Yes | Yes | Yes | Yes | Yes | Yes |
| Ataro et al. (2020) | Yes | Yes | Yes | Yes | Yes | Yes | Yes | Yes |
| Bune et al. (2020) | Yes | Yes | Yes | Yes | Yes | Yes | Yes | Yes |
