# Supplementary material for: Metabolic syndrome among people living with HIV in Ethiopia: a systematic review and meta-analysis
Source: Diabetol Metab Syndr. 2023 Mar 28;15:61. doi: 10.1186/s13098-023-01034-9 (PMC10045608; doi:10.1186/s13098-023-01034-9)
Supplement: Supplementary file 2 — Supplementary Material 2 [file 13098_2023_1034_MOESM2_ESM.docx]

**PubMed search strategy**

Population: People living with HIV

Exposure: HIV/AIDS

Comparator: factors

Outcome: Metabolic Syndrome

***CONCEPT 1:***

"Acquired Immunodeficiency Syndrome"[Mesh] OR HIV/AIDS[tiab] OR "HIV"[Mesh] OR "HIV positive"[tiab] OR "people live with HIV"[tiab]

***CONCEPT 2:***

"Metabolic Syndrome"[Mesh] OR "Metabolic syndrome"[tiab] OR "cardiometabolic syndrome"[tiab] OR "Metabolic Diseases"[Mesh]

***CONCEPT 3:***

Ethiopia
