# Supplementary material for: Metabolic syndrome among people living with HIV in Ethiopia: a systematic review and meta-analysis
Source: Diabetol Metab Syndr. 2023 Mar 28;15:61. doi: 10.1186/s13098-023-01034-9 (PMC10045608; doi:10.1186/s13098-023-01034-9)
Supplement: Supplementary file 5 — Supplementary Material 5 [file 13098_2023_1034_MOESM5_ESM.docx]

Figure 1: subgroup analysis by region

Figure 2: subgroup analysis by sampling technique
